# An ICT infrastructure to integrate clinical and molecular data in oncology research

**DOI:** 10.1186/1471-2105-13-S4-S5

**Published:** 2012-03-28

**Authors:** Daniele Segagni, Valentina Tibollo, Arianna Dagliati, Alberto Zambelli, Silvia G Priori, Riccardo Bellazzi

**Affiliations:** 1 IRCCS Fondazione Salvatore Maugeri, Pavia, 27100, Italy; 2 Istituto Universitario di Studi Superiori, Pavia, 27100, Italy; 3 Università di Pavia, Dipartimento di Informatica e Sistemistica, Pavia, 27100, Italy

## Abstract

**Background:**

The ONCO-i2b2 platform is a bioinformatics tool designed to integrate clinical and research data and support translational research in oncology. It is implemented by the University of Pavia and the IRCCS Fondazione Maugeri hospital (FSM), and grounded on the software developed by the Informatics for Integrating Biology and the Bedside (i2b2) research center. I2b2 has delivered an open source suite based on a data warehouse, which is efficiently interrogated to find sets of interesting patients through a query tool interface.

**Methods:**

Onco-i2b2 integrates data coming from multiple sources and allows the users to jointly query them. I2b2 data are then stored in a data warehouse, where facts are hierarchically structured as ontologies. Onco-i2b2 gathers data from the FSM pathology unit (PU) database and from the hospital biobank and merges them with the clinical information from the hospital information system.

Our main effort was to provide a robust integrated research environment, giving a particular emphasis to the integration process and facing different challenges, consecutively listed: biospecimen samples privacy and anonymization; synchronization of the biobank database with the i2b2 data warehouse through a series of Extract, Transform, Load (ETL) operations; development and integration of a Natural Language Processing (NLP) module, to retrieve coded information, such as SNOMED terms and malignant tumors (TNM) classifications, and clinical tests results from unstructured medical records. Furthermore, we have developed an internal SNOMED ontology rested on the NCBO BioPortal web services.

**Results:**

Onco-i2b2 manages data of more than 6,500 patients with breast cancer diagnosis collected between 2001 and 2011 (over 390 of them have at least one biological sample in the cancer biobank), more than 47,000 visits and 96,000 observations over 960 medical concepts.

**Conclusions:**

Onco-i2b2 is a concrete example of how integrated Information and Communication Technology architecture can be implemented to support translational research. The next steps of our project will involve the extension of its capabilities by implementing new plug-in devoted to bioinformatics data analysis as well as a temporal query module.

## Background

In the last few years, a noteworthy effort has been devoted to develop new Information and Communication Technology (ICT) infrastructures to support biomedical research. Such infrastructures have often been designed to integrate clinical and research data, in order to accelerate both the process of new discoveries in basic research, thanks to the rich phenotype information contained in clinical records, and the translation of research results in clinical practice.

In particular, this is the aim of the Informatics for Integrating Biology and the Bedside (i2b2) research center: i2b2 [1,2] is one of the seven centers funded by the NIH Roadmap National Centers for Biomedical Computing [3]. Among other important results, i2b2 has delivered an open source suite, which provides clinical investigators with a number of tools necessary to effectively perform the integration of clinical and biomedical records. I2b2 is based on a data warehouse, which is efficiently interrogated to find sets of interesting patients preserving their privacy through a query tool interface. Within this architecture, interoperable server-side software objects, called "cells", are able to exchange information with each other, relying on web services technology. The collection of all cells is named "hive" [4].

In order to support and improve the efficiency of clinical research in oncology, the University of Pavia and the IRCCS Fondazione Salvatore Maugeri of Pavia are developing and implementing a novel ICT platform, called Onco-i2b2, grounded on the i2b2 software.

Onco-i2b2 is able to integrate data from different sources inside the i2b2 data warehouse through the implementation of a complex IT architecture, which includes development of new i2b2-cells for data analysis. As result of this project, hospital researchers have been enabled to obtain information from the PU database, from a biobank management system and to merge them with the clinical information present in the hospital information system (HIS), in order to select interesting patients with a specific phenotype of interest.

Currently, the project manages patients with a principal diagnosis of breast cancer. However, since the oncology unit and the biobank of the FSM hospital are already equipped to handle information about samples coming from other wards or other European medical centers, new type of cancers will be considered in the near future.

## Methods

In this section we will describe the design principles and the implementation of the IT architecture that has been deployed at the FSM hospital within the Onco-i2b2 project. As mentioned in the previous section, our system is based on the i2b2 open source software environment, and its main features are briefly summarized below.

### An overview of the i2b2 technological solutions

The i2b2 framework has two main pillars: the data warehouse and the data management services [4].

The data warehouse is called Clinical Research Chart (CRC). The CRC is a based on a star schema [5], one of the fundamental data warehouse models. The CRC has a central "fact" table, called "observation fact", and four "dimension" tables, which represent "patients", "medical concepts", "visits" and "care providers". The observation table contains the patient data while the dimension tables contain further descriptive and analytical information about attributes in the observation table. A dimension table may contain information about how certain data is organized, such as a hierarchy that can be used to categorize or summarize the data. Such hierarchies are exploited to query the data and are referred to as the "i2b2 ontology" [6].

The data management services are divided into server-side software modules and client-side applications. The server-side software objects are called "cells" and they compose the "hive" of i2b2. Each cell is a web-service that communicates with the other cells through XML messages following REST standard [7] over HTTP. The current i2b2 release provides five necessary cells that make up the core of the i2b2 hive: File and Data Repository, Security, Ontology, and Identity Management. Currently, two client-side applications are available, a web-browser access which leverages on Javascript functions and a desktop Java application, the workbench, based on the Eclipse platform [4]. Both clients allow the user to fast query the data by dragging and dropping query concepts from the ontology (i.e. the previously described hierarchy of the dimensions), which is properly displayed. The concepts can be combined using an "and/or" mode and the query results are saved and may be exported to other client data analysis modules, which can be included as "plug-ins". Thanks to the technology developed within i2b2 it is possible to integrate information coming from different sources and collected for different purposes, in order to enable researchers to query and analyze the huge amount of data coming from the clinical practice and to plann further "in silico", "in vitro" or "in vivo" investigations.

### The Onco-i2b2 system

The main goal of the Onco-i2b2 project is to gather data and samples, which are collected during the day-by-day activity of the Oncology I department of the FSM, and to make them available for research purposes in an easy, secure and anonymized way. Once a patient is hospitalized, he/she may sign an informed consent to donate samples, specimens and data collected for clinical reasons to research. When this happens, the selected samples, coming from the PU unit, are anonymized and stored in the FSM biobank. At the same time, the data of the FSM HIS are collected and matched with the biobank samples, and stored in the i2b2 CRC.

Given the project purposes and needs, we had to develop a customization of the i2b2 architecture. In particular, in order to create a robust and safe integrated environment we had to face different types of problems.

#### 1. Privacy and anonymization

One of the most important issues we addressed is related to ensuring the anonymity of data processed and the need to avoid loss of information while we handle different types of data sources [8]. After considering different solutions [9], in order to ensure de-identification of biobank samples, we chose to anonymize each cancer biospecimen coming from the AP unit by creating a novel two-dimensional DataMatrix barcode, which does not include any direct reference to the donor patient and to implement an automatic procedure to populate the biobank database with some of the most interesting information contained in the pathology unit database. The relation between the pathology unit patient identifier and the novel identifier associated with biobank samples is saved in a lookup table stored on a database server located in a dedicated environment.

#### 2. Synchronization of the biobank database with i2b2

**Figure 1:**
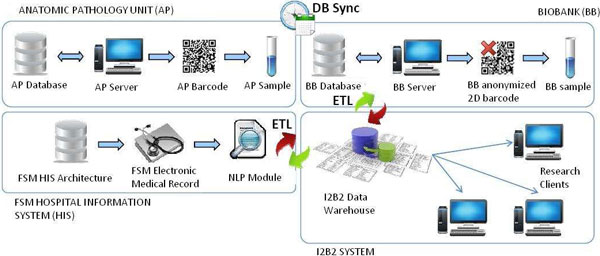
**FSM ICT architecture**. The ICT architecture designed to integrate information from the FSM PU, the HIS, the biobank database and the i2b2 system. Synchronization between de PU and the BB databases occurs periodically every fifteen minutes from 8am to 10pm. As backup procedure, a differential dump is made every day at midnight and files were stored on separated servers. The anonymization task is performed during this synchronization process. In the synchronization phase clinical information is retrieved from the FSM HIS for every patient that had at least one contact with the PU unit. A further ETL process merges all the information once a week and populates the Onco-i2b2 data warehouse.

The biobank database and the AP database are periodically synchronized during the day in order to keep biobank samples data constantly updated. Figure 1 explain in detail the synchronization process. The information on the biological samples contained in a biobank are then loaded into the i2b2 data warehouse through a series of Extract, Transform, Load operations that involve data extraction, processing and mapping in the data warehouse. The ETL activity was performed relying on the KETTLE software developed within the Pentaho project [10,11]. Figure 1 (points 1 and 2) shows the information flow described above. To facilitate the data entry task related to the day-by-day biobank data management and to reduce the risks of human errors, a dedicated web-based software was implemented. This software, developed in Java using the GWT (Google Web Toolkit) [12] libraries, allows the researcher to keep under control the information flow between the PU database and the biobank database. Before a novel bio sample coming from the PU unit is stored in the biobank, a number of preparation steps needs to be performed. All the information regarding these operations are stored using this software. Granted users may retrieve the information related to the donors' and patient's informed consent. In addition it is possible to manage sets of bio samples, categorizing them through various meta- attributes like project, type, creation date or description. These meta-data are also included in the ETL process and represents additional information available in the i2b2 system. Finally, the IT architecture relies on an application devoted to keep track of the biobank samples' locations; this is a third-party web based software that provides useful sample information such as fridge position, type of material, permissions related to the patient informed consent. This information is uploaded in the i2b2 data warehouse and made visible to the research through a dedicated web plug-in in the i2b2 web client, called Biobank Info.

#### 3. The i2b2 web client

The i2b2 web client is a web-based interface to the i2b2 hive, which reproduces much of the functionality of the " workbench" desktop client through a web browser [4]. The primary advantage of the web client is that software does not have to be installed on users' computers. This allows having an always-updated environment, reducing maintenance and immediately making available any new features to all users.

As previously mentioned, the information related to bio samples can be easily retrived by possible running the Biobank Info plug-in, located inside the analysis tool web page and totally compliant with the i2b2 1.5 version. By using this plug-in, the researcher can directly access the biobank samples information related to specific patient sets previously extracted with ad-hoc queries.

**Figure 2:**
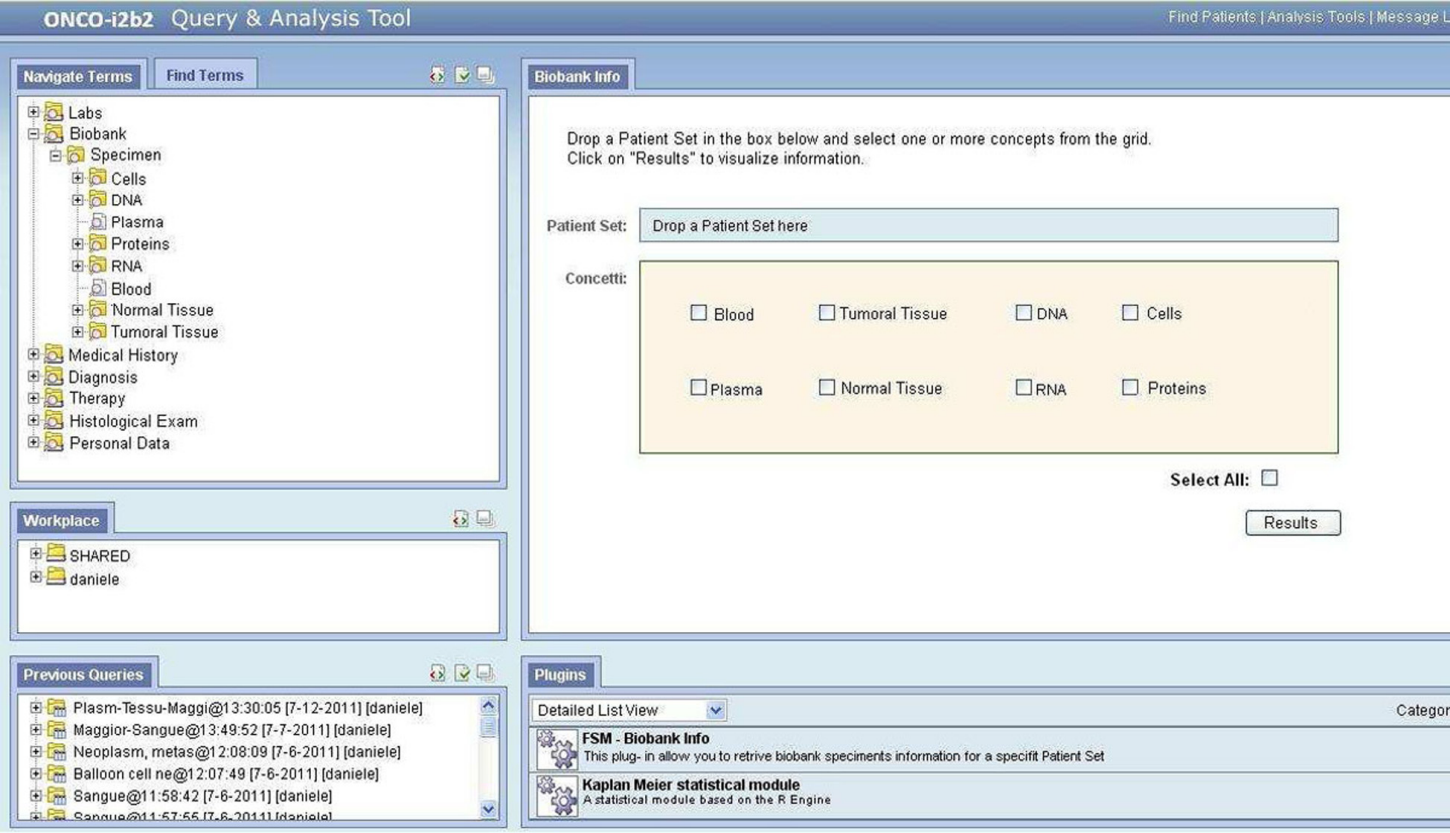
**Biobank Info plug-in**. The Onco-i2b2 Biobank Info plug-in, running on the i2b2 web client application. Using this tailored plug-in is possible to select which type of biosample is related to one defined patient set.

After dragging and dropping a patient set into the Biobank Info plug-in dedicated box (see Figure 2 for visible details), the user has to select the type of material contained in the samples to be searched (possible choices are visible in Figure 2) in order to view the requested information.

#### 4. Integration of i2b2 with the HIS and the NLP module

The main focus of the Onco-i2b2 project is currently dealing with breast cancer patients. Onco-i2b2 starts working when a biopsy is performed to obtain the detailed diagnosis. The tissue is tested in the pathology lab and a report is generated. The report contains the cancer diagnosis including the cancer 'stages' or the size of the cancer region.

Since these reports are only available in textual format, we developed a NLP software module aimed at extracting diagnostic information for all cancer patient health records that have at least one biological sample stored in the biobank. A large number of applications of the NLP technology has been presented in the context of the i2b2 project, and a specific cell has been developed, too. See for example [13]. Since the report is generated automatically as a result of a data entry process made by the PU unit, it has a pre-defined basic structure and contains coded texts such as SNOMED [14] terms related to tumor cells, tissues and organs topology and morphology. Other coded information automatically retrieved by our NLP module are related to the TNM [15], which contains information about the size of the tumor, whether the tumor has invaded the nearby tissues, the regional lymph nodes that are involved, the distant metastasis and the grade of the cancer cells.

The NLP software also retrieves clinical information related to specific medical tests such as the DAKO HercepTest, which measures the HER-2 (Human Epidermal growth factor Receptor 2 or c-erbB-2 oncoprotein, a protein related to breast cancer aggressiveness [16]) and some hormone receptors, like the estrogens receptors and progesterone receptors. Positive or negative results drive the classification of abnormal cells/tissues and provide a basis for the specific treatment selection. Finally, the NLP module also retrieves information regarding the Ki-67 expression [17]. Ki-67 is a cancer antigen that is expressed during cell growth and division, but is absent in the cell resting phase. The researchers agreed that a high level of Ki-67 is a biomarker related to aggressive tumors with poor prognosis. In fact, in a recent paper [17] it was found that breast cancers could vary in terms of hormone-sensitivity, lymph node status (positive or negative), but if the tumor has high levels of Ki-67, the risk of recurrence was higher than average.

Our NLP tool is based on the GATE system [18], thus following a software pipeline, in which each component has different settings and roles. One of the most important components is the sectionizer, which allows dividing the parsing document in categorized sections. We have configured this component in order to search only the coded part of the medical report, omitting the parts related to description and the patient's anamnesis. Once the correct section of the document is selected, the textual analysis is performed with the aim of finding the possible SNOMED codes, as well as the biomarkers described above. The software module that perform this task, based on GATE libraries, relies on a configuration file that contains the regular expression needed to find the SNOMED codes and the biomarker values, as well as a database containing all the SNOMED codes and their description. As a result of the entire NLP process, an XML file is generated containing a structured list of coded values ready to be imported in the i2b2 data warehouse. See the "Additional file 1" for the XML schema of the generated document, "Additional file 2" for the example of a real medical report and "Additional file 3" for the XML resulted from the NLP software module.

The system has been internally validated by a manual verification by the medical experts involved in the study on a subset of 100 cases with 100% accuracy. This module is now a crucial part of the entire software architecture.

#### 5. Ontologies and the NCBO Bioportal

As mentioned in the overview of the i2b2 technological solutions, the query interface relies on the definition of a set of hierarchies of concepts, mapped in the concept dimension table of the CRC; such hierarchies are referred to as the i2b2 "ontology". In order to cope with the needs of Onco-i2b2 we have composed the ontology by including both portions of pre-defined ontologies and a purposively defined hierarchy of concepts that maps the information contained in the HIS and in the biobank DB (i.e. a "internal" ontology).

As concerns the pre-defined ontologies, we wished to represent information related to SNOMED codes in a standardized way. For this reason, we relied on the NCBO BioPortal [19] web services in order to populate the i2b2 concepts data warehouse dimension. The strategy we adopted was to pull data from NCBO via dedicated REST web services and then reorganize the results into the format used by the i2b2 ontology cell.

The first step involved the selection of the ontology named "SNOMED morphologically Abnormal Structure" (Ontology Id 2058), a sub-tree of the SNOMED CT ontology that properly maps the information we needed. Once the BioPortal registration was successfully obtained, we were able to use the "View Extraction" web service, which extracts a branch of an ontology given a root term by iterating over all terms of a sub-tree. We use as root term a subset of the sub-tree that identifies neoplasm and hamartoma. The NCBO web service generates a Web Ontology Language (OWL) file format [20].

Using the ontology OWL file [21], through a Java based procedure we have processed the information in order to generate a data structure compliant with the i2b2 ontology cell.

**Figure 3:**
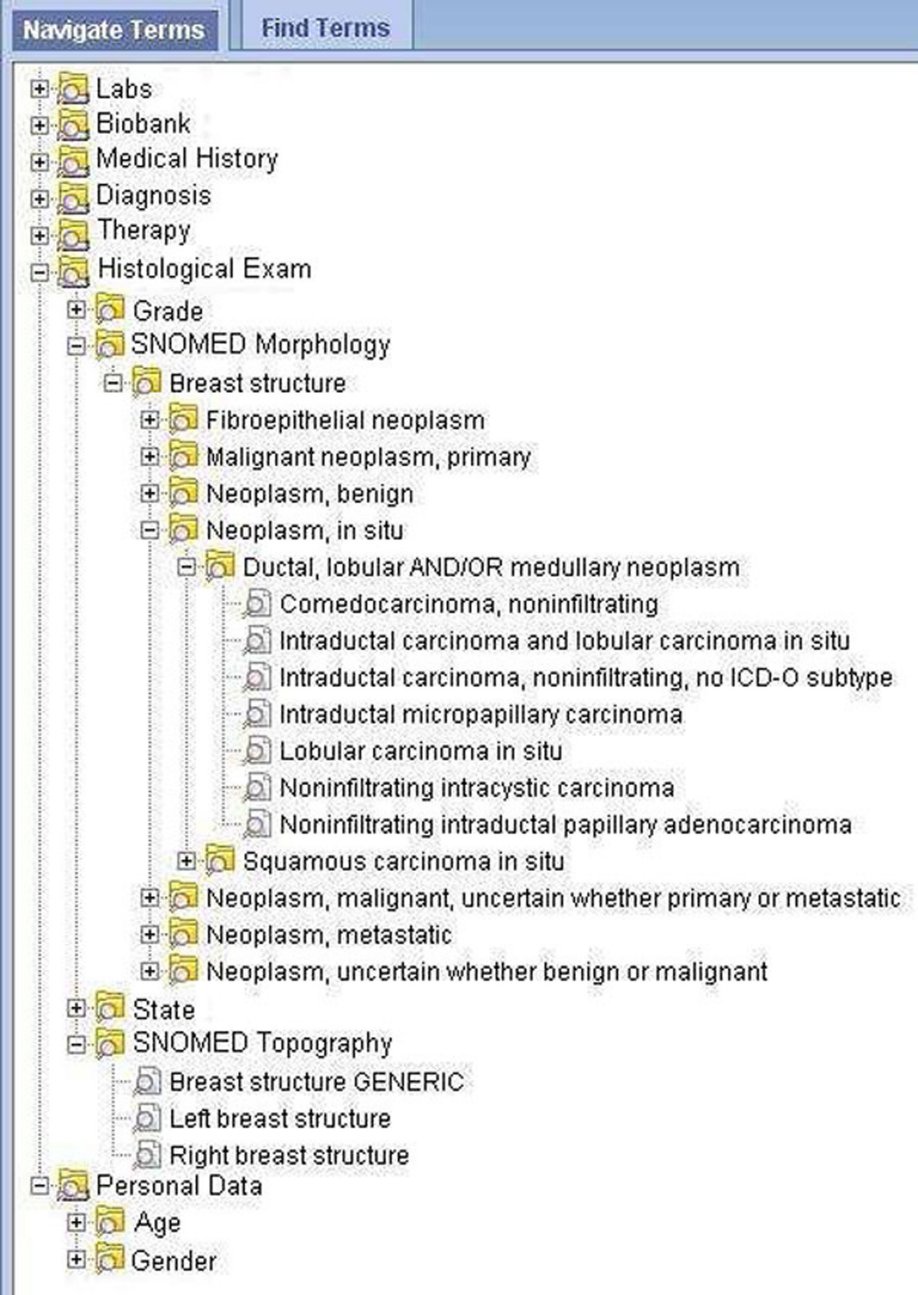
**Onco-i2b2 ontology**. The Onco-i2b2 ontology hierarchy. The figure highlights the section related to breast cancer SNOMED codes.

Figure 3 shows the ONCO-i2b2 ontology and the hierarchy related to the SNOMED concepts.

## Results

The main result of our work is an integrated ICT infrastructure that leverages on i2b2 to navigate information related to samples collected in biobank together with data derived from clinical practice; this system allows the FSM researchers to exploit i2b2 query capabilities relying on the user-friendly web interface available. We also extended the i2b2 web plug-ins with a tool that can manage information about patients bio samples.

After a four months period of trial and testing (December 2010- March 2011) the system is now up and running and the FSM hospital.

Currently, the i2b2 instance installed in FSM consists of 6,713 patients related to breast cancer diagnosis (393 of them have at least one biological sample in the cancer biobank since December 2010 that is the biobank operating start date), 47,140 visits, and 96,506 observations over 960 concepts.

In an effort to continuously improve this i2b2 based integrated architecture for hospital researchers, our team has already added two other novel web plug-ins for data export and for Kaplan-Meier survival analysis [22].

## Discussion

The experience gained during on with the Onco-i2b2 project is likely to have an impact in terms of results exploitation and generalization of the platform.

First of all, i2b2 is a steadily growing and very active community of open source software developers and users. Nowadays, more than 50 centers are part of the i2b2 academic user community around the world. The software developed within Onco-i2b2 is made available to this wide community and potentially used by the other groups. In particular, the previously mentioned data export plugin and an R-engine cell for data analysis have been shared in the community software repository and downloaded by other groups [23].

Moreover, Onco-i2b2 deals with the important problem of managing the integration of biobank data and clinical information, relying on a set of intertwined modules with different "concerns". By properly adapting the ETL layer, it would be possible to reuse the most part of the developed solution, with particular reference to the interface with the biobank database, the CRC, the client and the plug-ins. The Onco-i2b2 system can be thus adapted to other research centers dealing with cancer patients' data, with the need to integrate biobank samples information and HIS clinical data. As a matter of fact, a very interesting research direction is represented by the Shrine project [24], which aims to define a cross-institutional collaboration framework to share data with the i2b2 infrastructure. Onco-i2b2 would perfectly fit in this initiative, also allowing the management of samples in the biobank coming from different hospitals and research groups.

Further generalization can involve the extension of the proposed architecture to integrate data from patients suffering from different diseases. As a matter of fact, the FSM biobank has been recently upgraded to store blood samples coming from a variety of clinical departments, including oncology (for other types of cancer patients), nephrology, and pneumology.

## Conclusions

The design and implementation of an integrated ICT architecture that is able to merge information related to different hospital data sources and that can be useful for medical practice and research is a stimulating challenge.

The novel IT architecture created at FSM is a concrete example of how this type of integration can be correctly implemented and made available for scientific research. To achieve this goal we had to face several challenges: i) the development of a specific software that allows automatic upload of the information from the PU to the biobank database; ii) the generation of new anonymized barcodes when the biosamples are stored in the biobank; iii) the design, development and configuration of an NLP software module to extract information from unstructured medical report generated from the PU and containing information relevant to the clinical characterization of patients in cancer research; iv) the creation of dedicated ETL transformations to populate the i2b2 data warehouse with concepts related to cancer research. Each ETL process used is a reusable component that can be scheduled to perform different data transformation jobs, once the system will have to deal with new samples coming from other hospital wards, or from other hospitals departments of the FSM corporate network. In a similar manner, the NLP module was designed to be as modular as possible, to allow its reuse on other medical reports.

Exploiting the potential of this IT architecture, the next steps of the project will concern the extension of the data set imported by the HIS as well as the management of other data from the laboratory tests. We also plan to continue extending the capabilities of the Onco-i2b2 architecture by implementing new plug-in devoted to data analysis; in particular, we are working on an extension of the i2b2 query engine by adding temporal query capabilities.

Following the example of the experience reported in [25], the future developments of the project regard the integration of patient's genotype data, including Next Generation Sequencing derived information, which will require careful evaluation both in terms of the data representation and storage and of data security and privacy.

## List of abbreviations used

FSM: Fondazione Salvatore Maugeri hospital; I2b2: Informatics for Integrating Biology and the Bedside; ETL: Extract, Transform, Load; NLP: Natural Language Processing; TNM: TNM Classification of Malignant Tumours; ICT: Information and Communication Technology; HIS: Hospital Information system; CRC: Clinical Research chart; GWT: Google Web Toolkit; OWL: Ontology Web Language.

## Competing interests

The authors declare that they have no competing interests.

## Authors' contributions

DS: participated in the ICT architecture design and implementation, software and integration process design, ETL processes tests and NLP module tests. Drafted the manuscript.

VT: participated in the project software development, NLP module creation and ETL processes generation.

AD: participated in the ETL processes creation and in the ontology definition. Revised the manuscript.

AZ: participated in the project design, in the information flow implementation and in the ontology creation.

SP: participated in the project design and coordination.

RB: conceived of the architecture design, participated in the project functional analysis and coordination; being the principal investigator of the Onco-i2b2 project; revised the manuscript.

## Supplementary Material

Additional file 1 **XML Schema for the NLP module output file**. PDF file that represents the XML Schema used to generate the XML result file for the NLP module. Detailed below are the tags used: <document> is the xml root container; <name> represents the anonymized report id; <date> is the report date; <type> inform about histological or cytological type; <number> is the specimen number related to the report; <estrogens_receptors>, <progesterone_recoptors>, <Ki67>, <c-erb_B2> are related to laboratory analysis values, <grade> is the tumor grade, <state> in the TNM tumos code and <snomed> is a list of <code> and <name> that represent SNOMED values. Click here for file

Additional file 2 **Medical report example used with the NLP module**. PDF file that represents a real example of medical records used as input for the developed NLP software module. Click here for file

Additional file 3 **XML example for the NLP module output file**. PDF file that represents the XML format result file of the NLP module. The explanation of each tag is detailed in the description of "Additional file 1". Click here for file
