# Supplementary material for: An ICT infrastructure to integrate clinical and molecular data in oncology research
Source: BMC Bioinformatics. 2012 Mar 28;13(Suppl 4):S5. doi: 10.1186/1471-2105-13-S4-S5 (PMC3303735; doi:10.1186/1471-2105-13-S4-S5)
Supplement: Additional file 1 — XML Schema for the NLP module output file. PDF file that represents the XML Schema used to generate the XML result file for the NLP module. Detailed below are the tags used: is the xml root container; represents the anonymized report id; is the report date; inform about histological or cytological type; is the specimen number related to the report; , , , are related to laboratory analysis values, is the tumor grade, in the TNM tumos code and is a list of and that represent SNOMED values. [file 1471-2105-13-S4-S5-S1.pdf]

```
<?xml version="1.0" encoding="ISO-8859-1" ?>
<xs:schema xmlns:xs="http://www.w3.org/2001/XMLSchema">

<xs:element name="document">
  <xs:complexType>
    <xs:sequence>
      <xs:element name="name" type="xs:string"/>
      <xs:element name="date" type="xs:date"/>
      <xs:element name="type" type="xs:string"/>
      <xs:element name="number" type="xs:string"/>
      <xs:element name="analysis" type="analysisValues" minOccurs="0"
      maxOccurs="1"/>
      <xs:element name="grade" type="xs:string"/>
      <xs:element name="state" type="xs:string"/>
      <xs:element name ="snomed_codes">
        <xs:complexType>
          <xs:sequence>
            <xs:element name="snomed" type="snomedValues" minOccurs="0"
            maxOccurs="unbounded"/>
          </xs:sequence>
        </xs:complexType>
      </xs:element>
    </xs:sequence>
  </xs:complexType>
</xs:element>

<xs:element name="analysisValues">
  <xs:complexType>
    <xs:sequence>
      <xs:element name="estrogens_receptors" type="xs:string"/>
      <xs:element name="progesterone_receptors" type="xs:string"/>
      <xs:element name="Ki67" type="xs:string"/>
      <xs:element name="c-erb_B2" type="xs:string"/>
    </xs:sequence>
  </xs:complexType>
</xs:element>

<xs:complexType name="snomedValues">
  <xs:sequence>
    <xs:element name="code1" type="xs:string"/>
    <xs:element name="code2" type="xs:string"/>
    <xs:element name="name1" type="xs:string"/>
    <xs:element name="name2" type="xs:string"/>
  </xs:sequence>
  <xs:attribute name="itemId" type="xs:boolean" />
</xs:complexType>

</xs:schema>
```
