# Supplementary material for: An ICT infrastructure to integrate clinical and molecular data in oncology research
Source: BMC Bioinformatics. 2012 Mar 28;13(Suppl 4):S5. doi: 10.1186/1471-2105-13-S4-S5 (PMC3303735; doi:10.1186/1471-2105-13-S4-S5)
Supplement: Additional file 2 — Medical report example used with the NLP module. PDF file that represents a real example of medical records used as input for the developed NLP software module. [file 1471-2105-13-S4-S5-S2.pdf]

Tipo esame : Istologico N° Esame : 11-I-03276 Data accettazione : 08/06/2011

Reparto di provenienza : U.O. Chirurgia Gen. Senologia

Materiale inviato
A LINFONODO SENTINELLA CAVO ASCELLARE DESTRO INVIATO IL 08 06 2011 PER ESAME ESTEMPORANEO
B QUADRANTE SUPERIORE MAMMELLA DESTRA
C AMPLIAMENTO MARGINE SUPERFICIALE MAMMELLA DESTRA
D RADICALIZZAZIONE MARGINE PROFONDO MAMMELLA DESTRA
E RADICALIZZAZIONE MARGINE SUPERIORE MAMMELLA DESTRA
F RADICALIZZAZIONE MARGINE SUPERO ESTERNO MAMMELLA DESTRA
G RADICALIZZAZIONE MARGINE INFERO ESTERNO MAMMELLA DESTRA

Descrizione Macroscopica
A) Due linfonodi rispettivamente di cm. 1.6 e cm. 1.1 esminati completamente con sezioni multiple seriate.
B) Tessuto mammario di cm. 7 x 6 x 3, orientabile (peso gr. 73 dopo fissazione). Al taglio, prossima al margine
infero esterno (distanza minima cm. 1.2), neoplasia a margini infiltrativi di cm. 1.
C) Lembo cutaneo di cm. 5 x 0.5 x 0.3 incluso in toto.
D) Tessuto mammario del diametro maggiore di cm. 2.5 con filo di repere.
E) Tessuto mammario del diametro maggiore di cm. 2.2 con filo di repere.
F) Tessuto mammario del diametro maggiore di cm. 3 con filo di repere.
G) Tessuto mammario del diametro maggiore di cm. 2.5 con filo di repere.

Diagnosi intraoperatoria
A) Due linfonodi indenni da metastasi.

Reperti e Conclusioni
B) Carcinoma duttale infiltrante della mammella, G2 SBR, a crescita di tipo infiltrativo, con scarsa reazione linfoplasmacellulare
e minima componente (10%) peritumorale di carcinoma intraduttale di grado nucleare intermedio ed alto (DIN 2-DIN 3), prevalentemente di
tipo solido e cribroso con focale necrosi.
Assenza di invasione vascolare peritumorale.
Presenza di microcalcificazioni.
Involuzione adiposa nel tessuto mammario in esame.
Stato dei margini: indenni.
C) Lembo cutaneo indenne.
D) Tessuto mammario indenne.
E) Tessuto mammario indenne.
F) Tessuto mammario indenne.
G) Tessuto mammario indenne.

Valutazione assetto recettoriale, frazione proliferante e c-erbB2:
Recettori estrogeni: 90%
Recettori progesterone: 70%
Ki67: 15%
c-erb B2:punteggio DAKO HerceptTest: 3+ (positivo); intensa e completa colorazione delle membrane citoplasmatiche nell'80% delle cellule neoplastiche.

T-D8100 - M-09410
T-04000 - M-85003
T-04020 - M-09410
T-04000 - M-09410
T-04000 - M-09410
T-04000 - M-09410
T-04000 - M-09410

Stadio : pT1b-pN0(sn)-pM Grado: G 2
