# Supplementary material for: An ICT infrastructure to integrate clinical and molecular data in oncology research
Source: BMC Bioinformatics. 2012 Mar 28;13(Suppl 4):S5. doi: 10.1186/1471-2105-13-S4-S5 (PMC3303735; doi:10.1186/1471-2105-13-S4-S5)
Supplement: Additional file 3 — XML example for the NLP module output file. PDF file that represents the XML format result file of the NLP module. The explanation of each tag is detailed in the description of "Additional file 1". [file 1471-2105-13-S4-S5-S3.pdf]

```
<?xml version="1.0" encoding="UTF-8"?>
<document>
  <name>998000637_254080_254090.txt</name>
  <date>08/06/2011</date>
  <type>Istologico</type>
  <number>11-I-03276</number>
  <analysis>
    <estrogens_receptors>90%</estrogens_receptors>
    <progesterone_receptors>70%</progesterone_receptors>
    <Ki67>15%</Ki67>
    <c-erb_B2>3+</c-erb_B2>
  </analysis>
  <grade>G 2</grade>
  <state>pT1bÂ-pN0(sn)Â-pM</state>
  <snomed_codes>
    <snomed consistent="true">
      <code1>T-D8100</code1>
      <code2>M-09410</code2>
      <nome1>Axilla structure (body structure)</nome1>
      <nome2>No evidence of neoplasm (finding)</nome2>
    </snomed>
    <snomed consistent="true">
      <code1>T-04000</code1>
      <code2>M-85003</code2>
      <name1>Breast structure (body structure)</name1>
      <name2>Infiltrating duct carcinoma (morphologic abnormality)</name2>
    </snomed>
    <snomed consistent="true">
      <code1>T-04020</code1>
      <code2>M-09410</code2>
      <name1>Right breast structure (body structure)</name1>
      <name2>No evidence of neoplasm (finding)</name2>
    </snomed>
    <snomed consistent="true">
      <code1>T-04000</code1>
      <code2>M-09410</code2>
      <name1>Breast structure (body structure)</name1>
      <name2>No evidence of neoplasm (finding)</name2>
    </snomed>
    <snomed consistent="true">
      <code1>T-04000</code1>
      <code2>M-09410</code2>
      <name1>Breast structure (body structure)</name1>
      <name2>No evidence of neoplasm (finding)</name2>
    </snomed>
    <snomed consistent="true">
      <code1>T-04000</code1>
      <code2>M-09410</code2>
      <name1>Breast structure (body structure)</name1>
      <name2>No evidence of neoplasm (finding)</name2>
    </snomed>
    <snomed consistent="true">
      <code1>T-04000</code1>
      <code2>M-09410</code2>
      <name1>Breast structure (body structure)</name1>
      <name2>No evidence of neoplasm (finding)</name2>
    </snomed>
  </snomed_codes>
</document>
```
